# “Jewish History” beyond binary conceptions: Jewish performing musicians in Vienna around 1900

**DOI:** 10.1080/14725886.2017.1345189

**Published:** 2017-09-02

**Authors:** Klaus Hödl

**Affiliations:** ^a^ Center for Jewish Studies, University of Graz, Graz, Austria

**Keywords:** Jews, Vienna, similarity, performing musicians, Albert Hirsch, interaction, interconnectedness

## Abstract

The article draws attention to *similarity* as a new concept in cultural studies. Its novelty consists in the abrogation of binary classifications and concurrent retention of differences. *Similarity* may serve as an important category of analysis in Jewish Studies in that it focuses on commonalities without neglecting the differences between Jews and non-Jews. This will be demonstrated by its application to a conflict among Viennese *Volkssänger*, that is, performing musicians who sang *Wienerlieder* and played short burlesques, in the early twentieth century. The *Volkssänger* dominated Vienna’s popular entertainment culture throughout the nineteenth century. This situation started to change when the emerging film industry and variety theatres eclipsed the attraction of the *Volkssänger* for the Viennese population. They, in turn, attempted to preserve their share in the entertainment sector by lobbying the Austrian government to prevent Hungarian ensembles performing in Vienna. When Albert Hirsch, one of the most popular *Volkssänger*, also known for his anti-Hungarian sentiments, invited an ensemble from Budapest to Vienna, many of his colleagues felt betrayed. They retaliated against Hirsch by rendering his Jewishness an issue. Hirsch reacted by questioning Jewish and non-Jewish boundaries. He did so by relating an experience in which he, as a Jew, had perceived more common ground with Karl Lueger, Vienna’s anti-Semitic mayor, than with other Jewish *Volkssänger*. Hirsch’s somewhat odd behaviour can be made intelligible by employing similarity as an analytical concept.

At the turn of the twentieth century, Vienna was home to the third largest Jewish community in Europe, surpassed only by Warsaw and Budapest. The number of Jewish inhabitants had sharply grown over the preceding decades due to migration mainly from provinces of the Habsburg Empire. Up until the late 1870s, the Bohemian Crown Lands and Hungary provided the bulk of Jews moving westwards. From the late 1880s on, the preponderant majority of Jewish migrants were from Galicia and Bukovina (Pauley 1992, 24). Many Jews arriving in the monarchy’s capital had left their homes in search of a better life. Galician Jews, in particular, were among those who sought to escape miserable living conditions, lacking any prospect of improvement. Vienna was not always their primary destination. Many Jews first headed to urban centres nearest to their *shtetls*. Only if they failed to make a living there did they continue their journey onward to major cities. In keeping with this pattern, the Austrian capital was frequently their final alternative (Hödl 1999). Other Jews arrived in Vienna despite their plans to emigrate overseas. Due to various circumstances they made it only as far as the Austrian capital and decided to remain there.^1^ Cases of thwarted ambitions to travel to destinations other than Vienna were so notorious that they even became the topic of popular cultural productions.

One such frustrated effort is the theme of the song *Das jüdische Schaffnerlied,* which was very popular in the *fin-de-siècle* period. It was part of the profuse reservoir of so-called *Wienerlieder* (Viennese songs). *Das jüdische Schaffnerlied* was composed by Carl Lorens (1851–1909) and performed by Adolf Hirsch (1866–1931), a well-known Jewish musician.^2^ In the song a little boy from Tarnow in Galicia is sent by his father to Grosswardein, a town in Hungary (now Oradea in Romania). Unfortunately he boards the wrong train and to his surprise, arrives in Vienna instead. At first he wants to go back to Tarnow, but becomes trapped in Vienna. In the end, he manages to achieve a good living (Bohlman 2008, 161–164). The *Yingl* from Tarnow, a fictional character, represents the sector of Jewish migrants to Vienna who had no specific intention of moving and settling there, but who, thanks to a twist of fate en route, decided to stay.

Once in Vienna anti-Semitism made itself felt for many newcomers. Even if they did not experience it in a violent or verbally explicit way, it affected their lives. Anti-Jewish bias was most palpable in the field of employment. Jews could hardly gain a foothold in public service and were frequently discriminated against in the private sector. They fared best if they could establish themselves as independently employed.^3^ Consequently, many migrants took up the same occupation they had practised in Galicia, namely peddling. At the end of their long journey, they nonetheless found themselves in dire straits, their immediate circumstances scarcely better than the lives they had left behind. Newspapers carried repeated reports about desperate Jewish peddlers unable to feed their children and intent on suicide (see IWE 358, 31 September 1900, 2–3). Even the hope for future improvement could prove deceptive, since peddling fuelled anti-Jewish bias, restricting other occupational opportunities even further. Above all, it stirred anger among small shopkeepers and artisans, who feared the competition of these Jewish peddlers (Boyer 1981, 78).

In this context, a curious incident took place – curious in that it deviates from the historical accounts of rabid hostility against Jewish peddlers. One cold day in December 1896, Samuel Scholder, one of the many Jewish migrants to the Habsburg capital, was hawking toys on Rotenturmstrasse, in the very heart of Vienna. This irritated an employee of an adjacent store, who began to insult him. Soon the incensed aggressor attacked Scholder, punching and beating him. The brawl drew the attention of passers-by, who did not hesitate to intervene, coming to the aid of the Jewish victim. The aggressive employee suddenly found himself surrounded by an angry crowd and fled. A growing number chased after and captured the man, in their indignation physically accosting him. One man even broke his cane while striking the culprit over the head (IWE 23, 23 January 1897, 8).

## “Similarity” as a central thesis

This seemingly spontaneous manifestation of compassion for Scholder in his plight appears surprising. How can it be interpreted? Does the aid non-Jewish passers-by gave Scholder raise questions about the validity of a common assertion: was there general antipathy to Jewish peddlers? Does it suggest the presence of a bond between Jews and non-Jews that, for whatever reasons, has been overlooked by historians? Or was it simply a humane collective gesture in response to a gross act of misconduct against a defenseless person on the street that leaves the narrative of prevalent anti-Jewish resentment unchanged and can thus be ignored?

The employment of *similarity* as an analytical tool, the central thesis here can, I contend, help to explain this peculiar episode and other unusual events that deviate from predominant narratives. This will be shown following a more detailed explanation of the concept of *similarity* as applied here. In the paper’s second section, the notion of *similarity* is applied in order to explain an effort by Albert Hirsch, a Jewish performing musician, to find common ground between himself as a Jew and the anti-Semitic mayor Karl Lueger. In pointing out a commonality, Hirsch intended to transcend seemingly entrenched Jewish and non-Jewish boundaries. This section also provides an insight into a chapter of Jewish performing musicians that not yet been examined in the historiography on Viennese Jewry.

## The concept of similarity

Despite, as mentioned in the introduction, its pervasiveness in the Habsburg capital around 1900, anti-Semitism did not shape all social interactions between Jews and non-Jews. Some Jews, perhaps an appreciable number, maintained close friendships and other kinds of intimate contact with non-Jews (Geehr 2003, 111). Others were troubled by the broader anti-Jewish ambience, but never experienced discrimination personally. Any description of Viennese anti-Semitism, therefore, has to take its complexity and nuances into account. Yet as numerous as such amicable relations might have been, scholars generally do not question the seemingly fixed Jewish/non-Jewish divide. The conception of a deep rift between Jews and non-Jews remains entrenched in historiography. This comes paradigmatically to the fore in the frequently repeated assertion that Viennese Jews mingled among themselves and were only rarely invited to the homes of non-Jews (Lappin 2009, 35). There existed, in other words, insuperable boundaries between the Jewish and non-Jewish worlds, unchallenged by supposedly isolated instances of harmonious interaction.

Against this backdrop, the “Scholder incident” described above, which pitted a Jewish peddler and non-Jewish pedestrians against a non-Jewish aggressor, and other examples of solidarity between Jews and non-Jews, can only be comprehended as exceptions to otherwise divisive relations between them. As exceptions, they leave the accepted metanarrative of a Jewish and non-Jewish gulf untouched. The perseverance of this binary, despite instances to the contrary, is largely due to the use of heuristic concepts unsuited to transcend it; rather, they solidify the dualism.^4^ Even post-structuralist and post-colonial approaches to Jewish history, with their emphasis on concepts such as alterity and hybridity, imply a *fundamental* (Jewish/non-Jewish) gap (Osterhammel 2015, 78–79). *Difference* and *identity,* both indicative of this binary, have become the central catchwords in Jewish studies and historiography.^5^ There seems to be no interpretative framework within which amicable Jewish/non-Jewish interactions can be comprehended as acts taking place not *in spite of*, but *along with* anti-Jewish hostility, thereby bridging the chasm between Jews and non-Jews. However, this can be achieved by applying the concept of *similarity*.

*Similarity* is a fairly new concept in the wide realm of cultural studies.^6^ Although it was already reflected upon in the late nineteenth century, it has hardly played a role in research to date.^7^ This may be due to the fact that *similarity* is a very vague notion. Describing two people as similar means that they are somehow alike, but at the same time dissimilar (Koschorke 2015, 36; Bhatti 2016, 174). Many scholars perceive this lack of precision in the definition as problematic.

The employment of *similarity* does not usher in a new cultural turn. Rather, it aims to further develop given methodological approaches by re-conceptualizing familiar analytical instruments and applying a new perspective to the objects/subjects to be explored. Thinking in terms of *similarity* means not positing a principal contrast between two subjects (Assmann 2015, 169).^8^ The difference between *similarity* and other approaches in cultural studies that explore common ground and “mutuality” between two or more people can be highlighted by analysing the respective understanding of overlaps between two spheres of culture. In cultural studies, such realms of contact have been major topics in the recent past, as the ideas of *blurring boundaries* or *shared/entangled history* reflect (Schmale and Steer 2006; Rosman 2007; Penslar 2009, 10–14; Perry and Voß 2016, 1–13). To date they have always been, at least implicitly, conceived of as spaces where commonalities between otherwise clearly distinct cultures were *negotiated*. This process of negotiating implies a “self” and “other” who determine what they share, but concurrently leaves the dichotomy between them intact.^9^

According to the model of *similarity*, these overlaps merely provide conditions for the perception of connectedness, that is, similarity*.* There is no negotiation, solely a situational experience, with *identity* and *difference* no longer serving as reference points (Assmann 2015, 168).^10^ *Similarity* is thus not predetermined. The awareness of *similarity* results from encounters, from dynamic interactions as elaborated by Bruno Latour in his theory of actor-network (ANT) (Latour 2014). According to this model, the “social” is anything but given, but results from permanent contacts and associations, that is, the premises for experiencing *similarity*.

An integral component of the model of *similarity* is its acknowledgement of difference. In contrast to other postmodern approaches, *similarity* considers difference as vague rather than fundamental and profound. Difference comprises a dissimilarity that comes along in various shades and degrees, rather than as a stark contrast (Koschorke 2015, 36). Similarity and dissimilarity are thus gradations constituted by the angle of the observer, instead of substantial properties. Consequently, a study of similarities between Jews and non-Jews acknowledges, albeit non-antagonistically, certain Jewish distinctions (Langenohl 2015, 106).

*Similarity*’s simultaneous recognition of differences *as well as* commonalities may serve as the much-needed middle ground between the two apparently exclusive approaches around which the exploration of Jewish self-understanding usually revolves. Analysts either tend to essentialize Jewishness, thereby conceptualizing at least its core as pre-given and permanent.^11^ Or they deconstruct it in a way that risks dissolving its Jewish traces altogether (Rosman 2007, 4.). There is, for instance, a broad consensus among scholars that identity must be considered dynamic, processual and situative, that is, changing from context to context.^12^ Due to its fluid character it is difficult to describe and impossible to define. This understanding of identity, although compatible with largely uncontested findings in cultural studies and therefore very promising, bears the risk of rendering Jewishness elusive and unrecognizable (Burákova 2016). The analytical tool of *similarity*, by contrast, emphasizes features of (gradual) Jewish difference that tend neither to disappear due to the fluidity of Jewishness, nor to be essentialized.

Space has become a major topic in recent research in cultural studies. A wide array of literature exists especially on *hererotopia*, as Michel Foucault describes it (2014, 7–22) or *liminal space* according to Arnold van Gennep (1999, 27–28) and Victor Turner (2000). As fruitful as the use of these concepts in cultural studies may have been, the space pertaining to *similarity* may best be described as a so-called non-space (*non-lieu*). Marc Augé designates airports or hotels as *non-lieux* (Augé 2014). People tend to frequent them for very pragmatic reasons. In pursuance of their goals, visitors pass through, rather than familiarize themselves with such spaces, thereby recognizing basic similarities as well as differences between themselves and others. Beside this awareness they usually have no relationship to the people around them, nurture no interest in them and merely try to get along with them. They are conscious of distinctions, but do not attempt to understand them.

Similarity, as mentioned above, is an ephemeral experience. Whenever people become aware of their similarity with others, unusual alliances between them are possible. It can spark a sense of empathy transcending lines of distinction (Assmann 2015, 172). This is exactly what happened with the defence of Scholder by non-Jews: they pitied him at a moment of intense distress and expressed a feeling of solidarity with him that eclipsed any Jewish/non-Jewish antagonism. This is not to say that all differences receded. The compassion only indicated that in this particular situation, a sense of human connectedness that belies the idea of a Jewish and non-Jewish dichotomy prevailed. In other words, the “Scholder incident” is evidence of Jewish and non-Jewish relations beyond the dualism of sameness and otherness. Weaving such instances together would result in a narrative of the Jewish past that differs from the predominant historical accounts. It would not neglect anti-Semitism but provide a more comprehensive story of Jewish and non-Jewish relations. The question raised above, whether that incident must be comprehended as an exception to an entrenched Jewish and non-Jewish divide, should be answered in the negative. Neither was it the norm. Rather, it was part of a broad range of diverse Jewish and non-Jewish interactions.

Another puzzling example that can be rendered more intelligible by employing the concept of *similarity* concerns a conflict between Albert Hirsch (1841–1927), a renowned Jewish *Volkssänger*, that is, performing musician, and the majority of his Jewish and non-Jewish peers in Vienna shortly after the turn of the twentieth century. *Volkssänger* were the primary entertainers in Vienna in the second half of the nineteenth century before their popularity was eclipsed by the emergence of music halls, the movie industry and the cabaret. The *Volkssänger* sang Viennese songs (*Wienerlieder*) and performed short theatrical plays.

## The “war” among the Viennese *Volkssänger*

On 24 December 1901,^13^ Viennese newspapers announced a meeting of *Volkssänger* and performers scheduled to take place shortly after Christmas (IWE 353, 24 December 1901, 9).^14^ Readers of the notice must have had the impression that the forthcoming gathering was of the utmost urgency and therefore hurriedly convened. In fact, three days later, immediately after the Christmas holidays, the *Volkssänger* came together at the Goldener Luchs, an inn located in Ottakring, the 16th district in Vienna.

Considering Vienna’s cultural topography, the location of the meeting sheds light on the self-understanding of the *Volkssänger* and positions them in the city’s cultural landscape. Ottakring was a fairly new district on Vienna’s periphery. It belonged to an area called *Vorstadt.* The term designates neighbourhoods that had been independent villages before their incorporation into Vienna in 1892. In that year, the two localities of Ottakring and Neulerchenfeld were merged into the district of Ottakring (Leitner and Hamtil 2006, 7). The *Vorstadt* neighbourhoods differed in various ways from the older parts of the Habsburg capital. On the one hand, and this was particularly true for Ottakring, they served as an area where industrialization began to expand. On the other, as was the case with Neulerchenfeld, they also retained a rural ambience and village structure, where many inns were located for the performance of musicians, above all the *Volkssänger*. These musicians drew part of their audience from the labour force employed in the factories situated in nearby areas. Among these inns, for example, was the Stalehner, which later became one of the most important variety theatres in Vienna (IWE 53, 23 February 1907, 7).

Combined, the two characteristics of Ottakring, its rural ambience and large proletarian population, provided not only seminal ground for a thriving *Volkssänger* tradition, but also rendered the neighbourhood the opposite of the 1st district, *Innere Stadt*, with its imperial glamour and institutions of high culture, such as the opera house and the Burgtheater, as well as the residences of the nobility and higher bourgeoisie (Maderthaner and Musner 1999, 35, 118). Through their choice of the Goldener Luchs, the *Volkssänger* styled themselves as rooted in the suburban, low-brow cultural realm.

The meeting on 27 December 1901 attracted around a hundred people. They represented only a fraction of the overall number of performing musicians in Vienna.^15^ However, their most important representatives attended the event, and their presence lent the gathering an element of particular importance. This impression was furthered by the fact that Karl Spacek (1850–1904), one of the most renowned Viennese *Volkssänger* of the day^16^ had organized the gathering. The reason for the gathering was a ban enacted by the Hungarian authorities on a Viennese *Volkssänger* group intending to perform in Budapest (Gluck 2016, 139–178). Those participating in the meeting were to discuss reactions to this prohibition. The agenda was announced in advance and was not expected to cause any serious disputes. It was, therefore, surprising that the meeting turned into a forum for various arguments, spite, insinuations and much commotion. An exploration of the disruptive discussions provides an instructive insight into the circumstances under which the *Volkssänger* had to work, the most urgent problems they had to tackle in pursuit of their profession, and the poverty with which many of them had to struggle. In addition, it seems to emphasize a line of demarcation between Jewish and non-Jewish *Volkssänger*. One of the central questions I address here is whether anti-Jewish sentiments were inherent in the institution and culture of the *Volkssänger*, or if their articulation during the *Volkssänger war* was a deviation from basically harmonious relations between Jewish and non-Jewish *Volkssänger*?

### The meeting at the Goldener Luchs

The gathering in late December 1901 was dominated by speeches delivered by Karl Recher and Karl Rötzer (1862–1908).^17^ After short references to the “Budapest affair,” the ban on performances by Viennese *Volkssänger* in Hungary, the speakers both diverged from the actual reason for calling the meeting and touched upon various facets of the work of the *Volkssänger* in Vienna. Three issues were of particular relevance to the further development of the *Volkssänger* movement. The first relates to the social adversity in which many *Volkssänger* either lived or of which they were at risk. All the *Volkssänger* present at the meeting agreed that an improvement in their working conditions required more political activism. They decided to increase their political lobbying and attract the attention of politicians to the dire circumstances in which they lived.

The second point concerns a certain hostility towards Albert Hirsch, the Jewish director of the *Volkssänger* ensemble *Gesellschaft Hirsch*.^18^ The question of whether his Jewishness exacerbated, or even caused, the grudge some of the *Volkssänger* bore against him, or if their adverse feelings against him were independent of his being Jewish, cannot as yet be answered. This is only possible after an analysis of the entire *Volkssänger war*, which lasted until the spring of 1903. The first indication of this antipathy against Hirsch was implied in Recher’s speech in which he mentioned so-called *Damenkapellen*, namely musical bands consisting in the main of young women, which sometimes played between single acts of *Volkssänger* performances. In stating that their members were frequently employed despite an apparent lack of talent, and that they usually made a good living even though they earned very little, Recher insinuated an overlap between those women and prostitutes.^19^ In so doing, he broached something that had characterized the *Volkssängertum* from its very inception in the early nineteenth century (Hauenstein 1976, 63). The first female *Volkssängerinnen* were prostitutes before they joined the singing profession (Brauner-Berger 1993, 53). Even after their change of occupation, they frequently combined both jobs.

The blurring of boundaries between prostitutes and *Volkssängerinnen* was widely known in Vienna at the time. With his allusion to the *Damenkapellen*, Recher might have had other goals in mind than raising a new issue. He might have wished to disentangle the two occupations and revise the reputation of the *Volkssänge*r profession. Recher might also have intended to attack Hirsch. Since Hirsch was known for having a *Damenkapelle* perform during his ensemble’s performances, the issue Recher addressed implied censure of Hirsch without mentioning his name (IWE 183, 6 July 1900, 15).

Whereas it was not exactly clear whether Recher was speaking in general terms and criticizing Hirsch only implicitly, alongside others, or if his remarks were directed specifically at him, there was no doubt about the addressee when Amon Berg, another widely acknowledged representative of his profession^20^ joined the discussion. In his remarks, he questioned the sense of responsibility and moral attitude of those directors of *Volkssänger* ensembles who employed colleagues but invited guest performers to play on weekends. The *Volkssänger* under contract thus involuntarily forfeited part of their expected and much-needed income. Albert Hirsch belonged to the category of directors Berg criticized (see IWE 350, 21 December 1901, 15). Everybody at the meeting knew that Hirsch was the specific target of Berg’s expostulations. Hirsch tried to respond. At first, his attempts to justify his procedure were dismissed. Karl Spacek even called him a liar, thus adding fuel to the smouldering fire.

After the ensuing commotion had quietened down, Hirsch was formally given the floor to reply to his detractor. He did not linger over his response to Berg’s statements, reverting instead to the actual subject of the meeting by presenting the situation of Austrian *Volkssänger* in Hungary. He argued for retributive measures against Hungarian groups. He said that the Viennese *Volkssänger* should not relax until the last “Gypsy,” as he derogatively called Hungarians, had been expelled from the Austrian capital (IWE 356, 28 December 1901, 4). Hirsch championed a ban on Hungarian ensembles. In the succeeding months, becoming one of the staunchest supporters of this procedure, perhaps even the most rabid one. His unyielding attitude became one of his distinctive features.

The three components mentioned – the fight against the spectre of poverty permanently hovering over the *Volkssänger*, even the successful ones, a palpable, but not explicit hostility towards Hirsch, and his suggestion to prevent Hungarian groups from performing in Vienna – were to remain constants over the next few months. When Hirsch’s attitude towards Hungarian ensembles changed in early 1903, the entire structure of *Volkssänger* activism was transformed. The disputes among them almost degenerated into fisticuffs and violent brawls. At the same time, the xenophobic sentiments against Hungarians, which usually prevailed over any other adverse feeling, gave way to articulations of Judeophobia. Previously there had been little animosity among the *Volkssänger* towards their Jewish colleagues. At the Goldener Luchs, Recher even stated explicitly that the *Polnischen*, that is, Galician Jewish *Volkssänger* who performed in Vienna, were not considered to be foreigners and should not be affected by the ban against Hungarians. In other words, Austrian Jewish ensembles were to be excluded from the xenophobic measures (IWE 356, 28 December 1901, 4).

Recher’s reference to the *Polnischen* did not come out of the blue. A few days before the meeting of the *Volkssänger*, the police had raided some locales in the second district, Leopoldstadt, where Jewish immigrants preferred to settle and Galician groups performed (Hödl 1993). The official reason for the intervention was related to Yiddish, the language of their performances. According to the police, the use of Yiddish was not allowed in Vienna because the general public did not understand it (IWE 356, 26 December 1901, 6).

The actual reason for the raid, however, must be linked to Austrian censorship. Ensembles intending to perform were obliged to submit their pieces to the authorities in advance for examination and approval. They were allowed only to perform versions that had been cleared and released by the censor. The *Polnischen* appeared to abide by the law, yet an anonymous letter to the police insinuated that they performed plays deviating in content from the version they had given to the censor (IWE 356, 26 December 1901, 6). The police had to react to this accusation and carry out the raid.

Since the most important competitors of the *Polnischen* were other Jewish ensembles, it may well be that the letter was written by one of them. It is safe to assume that in his reference to the *Polnischen*, Recher intended to stress that the raid was an internal “Jewish affair” with which non-Jewish *Volkssänger* had no connection or concern.

### The attitude of the Volkssänger to modernization

Over the next 15 months, the Viennese *Volkssänger* met several times. Along with some other colleagues, Hirsch took over leadership functions of the burgeoning *Volkssänger* movement. He retained, above all, his profile as one of the prime instigators of anti-Hungarian sentiment. This may appear surprising. After all, large segments of Austrian society deemed Jews to be strangers, aliens, outsiders (Berkley 1988). So why did Hirsch, Jewish himself, try to distinguish himself from his colleagues by whipping up an anti-foreigner mood? Was Hirsch intent on proving his “Austrianness” by lashing out at a construed enemy?

To answer this question, it is necessary to put the two central goals of the *Volkssänger* into proper context. It is doubtful that the improvement in their working conditions and prohibiting Hungarian ensembles from playing in Vienna were the actual, not secondary, reasons for triggering their attempts to rectify their growing economic hardship. After all, bad working conditions had been distressing the *Volkssänger* for years. Moreover, the measures taken by the Hungarian authorities were certainly annoying, but did not affect the primary locus of work of the Viennese *Volkssänger*, the Austrian capital. So why, did the two objectives suddenly assume such a prominent place in the agenda of the performing musicians?

On closer examination, it becomes obvious that the goals of the *Volkssänger* were connected with their attitude to new developments in the entertainment sector. At the turn of the twentieth century, interest in the *Volkssänger* performance was dwindling among the Viennese population. New attractions, such as the emerging film industry and the increasing number of performances by internationally renowned artists in variety theatres, lured people away from the inns and locales where the *Volkssänger* played. In comparison to the striking artistry in the mushrooming music halls, the singing of Viennese songs appeared tedious to a growing number of people. Many immigrants tended to be, at best, indifferent to *Wienerlieder* and preferred to listen to the music of their home region. Slavs, for example, flocked to *tamburizza* concerts and preferred their musical traditions to Viennese songs.^21^ The *Volkssänger* found themselves in a virtual state of crisis as their audience was declining. Although they had faced problems as professional entertainers in former years as well, the new threat to their already dismal circumstances was the last straw. The *Volkssängers’* sudden activism can be understood as a frantic effort to forestall a further setback.

The *Volkssänger* had two options in reacting to their calamitous situation. They could either try to hold on to their shrinking share of the entertainment sector by restricting the access of potential competitors to their market; or hope to draw in new talent that would reform the entire Volkssänger profession. To put it briefly, they could choose the preservation of the status quo or modernization. The attitude towards the Hungarians was the litmus test. The *Volkssänger* obviously decided against overhauling their ever more outdated profession.

The lobbying to prevent Hungarian ensembles from playing in Vienna was therefore the performing musicians’ attempt to reserve for themselves the decreasing opportunities to perform, rather than retaliation for banning a Viennese group from playing in Budapest. The expression of hostility towards Hungarians was a means of achieving specific goals, rather than an end in itself. This was also the case with Albert Hirsch’s anti-Hungarian statements, as the further development of the *Volkssänger* movement reveals.

### Albert Hirsch’s self-revelation

In early 1903, a short note in a daily newspaper caused much confusion and turmoil among the *Volkssänger*, aggravated existing tensions, and finally led to the so-called war (*Volkssängerkrieg*). The note announced the move of the well-known ensemble *Folies-Caprice* from Budapest to Vienna and designated the location of its future performances as the Hotel Central on Taborstrasse, in the heart of Vienna’s Jewish Leopoldstadt district. The directors of three music halls adjacent to this venue protested against the transfer of the *Folies-Caprice*. These directors were Karl Lechner of the *Budapester Orpheumgesellschaft*, Fritz Lung of the *Folies-Comiques*, and Albert Hirsch, who was in charge of the *Lemberger Singspiel-Gesellschaft* at Edelhofer’s Leopoldstädter Volks-Orpheum.^22^ It was a particular irony of fate that Hirsch, the apparently xenophobic rabble-rouser, had to deal with a Hungarian troupe in his immediate surroundings.

The forthcoming arrival of the *Folies-Caprice* heightened nationalist sentiments. Since all three Viennese ensembles and the *Folies-Caprice* from Budapest were known as “Jewish groups,” it also had reverberations for internal Jewish relations. Although their audiences consisted of non-Jews as well, they primarily targeted Jews as spectators. Consequently, the *Folies-Caprice*, as might be expected*,* would above all poach on the Jewish preserve. The animosity against the new troupe was most severe among the Jewish *Volkssänger*. Therefore, the protest waged by Lechner, Lung and Hirsch rendered the forthcoming arrival of the Hungarian ensemble, at least in part, an internal Jewish affair.

A few days after this note in the press, an open letter appeared in the same newspaper, signed by Franz Pischkittl, the leaseholder of the Hotel Central, where the *Folies-Caprice* were scheduled to play, and Albert Hirsch, the actual author of the letter (IWE 74, 16 March 1903, 3). It raises several issues. In sum, the letter criticizes the *Volkssänger* movement and questions the ambiguous attitude of some of its leaders towards Hungarians. As Hirsch points out, there were two female singers from Hungary among Vienna’s *Volkssänger*. One of them had been engaged by Karl Recher, and the other had played until recently with the *Budapester Orpheumgesellschaft* headed by Lechner (*Das Variété* 20, 18 March 1903, n.p.).^23^ In public, however, both directors displayed an anti-Hungarian attitude.

Hirsch goes on to elaborate upon the *Budapester Orpheumgesellschaft*. He stresses that when they had set foot in Vienna in the late 1880s he lost his gainful engagement in the Leopoldstadt, but he did not protest (*Das Variété* 20; 18 March 1903, n.p.). The founding of new groups and the concomitant replacement of others, as he implies, was inherent to the very nature of the “*Volkssänger* business.” Although new, the “Budapester” quickly adapted to the Viennese milieu, representing an example of successful integration. Consequently, there was no need to be afraid of the *Folies-Caprice*. Within a short period, they also would become full-fledged Viennese.

After this attempt to placate his opponents, Hirsch reveals the actual reason for the open letter. He signs it “A. Hirsch, Viennese and potentially sole director of the new family-varieté at the ‘Hotel Central.’”^24^ In other words, only a few days after he had protested against the scheduled arrival of the *Folies-Caprice*, Hirsch introduced himself as the prospective director of the Hungarian ensemble. It was Hirsch who had provided the ensemble with the opportunity to perform in Vienna.

### Similarity through performance

Following his act of self-revelation, Hirsch faced severe hostility from a large segment of the *Volkssänger* community.^25^ They accused him of betrayal and egotism. It did not take long, however, before he was given the opportunity to respond formally to their criticism. This happened at a meeting the *Volkssänger* arranged at Seifert’s Saal, located in the Viennese district of Hernals. Hirsch’s imminent justification of his action aroused immense interest among his peers*.* They flocked to the scheduled gathering. When Hirsch arrived with some 20 supporters intent on backing him against his critics, the hall was already packed.

The convention was meant to temper the tensions among the *Volkssänger.* However, it clearly failed in that objective. Instead, it aggravated the already abrasive relations among them. As might be expected, the meeting started with an exchange of slanderous remarks, hurled at Hirsch, as well as cast by him at his detractors. The subsequent speeches by both Hirsch and some representatives of the performing musicians were also delivered in an aggressive, accusatory tone. Their analysis reveals more than mere grudges both parties bore against each other. They express anti-Semitic sentiments not openly articulated among the *Volkssänger* to this point, and provide an explanation for the antipathy displayed to Hirsch at the first meeting at the *Goldener Luchs* in December 1901.

Recher starts his remarks with a reference to Hirsch’s behaviour in the past. He reminds his colleagues that Hirsch had been double-crossing them for years. The issue about the *Folies-Caprice* was, Recher stated, only his latest act of cheating. Recher even provides an explanation for it. According to him, Hirsch was offered 12 gulden by the *Folies-Caprice* to serve as their director. Since this amount exceeded his earnings with th*e Lemberger Singspiel-Gesellschaft*, he readily agreed. Hirsch thus deceived the Viennese *Volkssänger* for the sum of a few gulden. Subsequently, Recher describes Hirsch as a person who changes his character as often as other people change their underwear. He always serves those from whom he benefits most (*Das Variété* 21, 25 March 1903, n.p.)

Recher does not stop at denigrating his adversary. He also touches upon the *Lemberger Singspiel-Gesellschaft*, referring to the members of the ensemble as “Polish Jews.” Although some people in the audience immediately interrupt and ask him to call them “Polish artists” instead of Jews, Recher reiterates the former designation (*Das Variété* 21, 25 March 1903, n.p.). He thus draws a line not only between Polish and Viennese, but also between Jews and *Volkssänger*, implying that Hirsch cannot be included among the latter.

Following Recher, Joseph Modl, himself Jewish, does not respond to Recher’s anti-Jewish references, but emphasizes that the *Budapester Orpheumgesellschaft*, whom Hirsch had termed a Hungarian ensemble in his open letter, consisted mostly of Viennese performers.

Hirsch then takes the floor. He endeavours to give an intelligible account of his actions. In so doing, his conception of identity as performative engagement comes to the fore. He starts by evoking the inauguration of the *Volkssänger* flag in October 1900, which was celebrated with a festive demonstration in Ottakring and the concluding consecration of the flag in a church (see IWE 283, 15 October 1900, 3). The festivities represented a constitutive event for the *Volkssänger*, strongly contributing to their sense of togetherness. Being absent from the inauguration may thus be understood as deliberately keeping one’s distance from the *Volkssänger*, not wanting to be considered their equal. Consequently, Hirsch emphasizes his participation in it. He reminds his colleagues that the *Budapester Orpheumgesellschaft* avoided the event, thus implying that its members showed less identification with the profession than he did.

Hirsch is not interested in publicly deprecating the conduct of the “Budapester.” Rather, his reference to them is meant as a retort to Recher’s polarization of Jews vs. non-Jewish artists. Hirsch points out that Jews cannot be conceived of as a monolithic group. Jews display different modes of behaviour, express different wishes and sentiments. Some, like Hirsch, lay great store on being included among the *Volkssänger*; others, such as the “Budapester,” do not. Jews cannot be essentialized.

According to Hirsch’s viewpoint, national or ethnic designation is not indicative of a person’s inner life or behaviour. Instead, people should be categorized according to their activities. Focusing on practices transcends construed lines of demarcation. He exemplifies his view by referring to a personal experience: during the consecration of the *Volkssänger* flag in the church, he came to stand beside Karl Lueger. Because he is a Jew, Hirsch asserts, he could not exclaim “All hail Lueger!” Still, he admits, it was nice of him to attend the ceremony, whereas the “Budapester” were absent.^26^ Hirsch thus finds Lueger likeable for his attendance at the event. For Hirsch, their shared participation in the inauguration establishes common ground between them that is missing between Hirsch und the Jewish members of the “Budapester.” At this particular moment, Hirsch feels a sense of *similarity* with an anti-Semite.

*Similarity* is a situational experience that does not extinguish all differences. Hirsch retained a feeling of otherness as he indicates in his explanation for not having praised Lueger. Yet this distinction was no longer fundamental. It was moderated by a concomitant sense of closeness and affinity.

Again, as was the case with the Jewish peddler Salomon Scholder, Hirsch’s experience of a common bond with, and feeling of respect for, Lueger should not be seen as an extraordinary emotion in an overly anti-Semitic climate. Rather, the sense of closeness existed side by side with Judeophobia. The mayor himself embodied this contrast: he used anti-Semitism for political gain but had Jewish friends and occasionally sided with Jews who faced anti-Jewish slander (Berkley 1988, 96). It is safe to assume that this ambivalence, solidarity and hostility, was deeply anchored in Viennese daily life. Otherwise, if Jews had only been suffering anti-Semitism, their existence in the city would have been unbearable. Admittedly, textual evidence of Jewish and non-Jewish unity is relatively rare compared with the abundance of anti-Semitic pamphlets and diatribes. This may be due to experiences of *similarity* being lived practices, not meant to be recorded. But retrievable they are, as the description of a soccer game between Hakoah and the non-Jewish team Brigittenauer A. C. in the 1920s demonstrates. Many non-Jews attending the event had a stake in, and were rooting for, Hakoah’s win. Since they had never cheered for Jewish players, the spectators did not know how to address them. One of the onlookers finally exclaimed: “*Hoppauf, Herr Jud*” (Torberg 2011, 59–68). As odd as this designation sounds, it indicates a relatedness between the non-Jewish soccer fan and a Jewish player. It is a situational experience, not preceded by any negotiating activities or interactions, with a sense of difference retained, as the shouting of “*Herr Jud*” as a form of address indicates.

### The end of the “war”

The meeting at Seifert’s Saal could not solve the dispute among the *Volkssänger.* Quite the contrary. Hirsch on the one side, and Recher, as well as Rötzer, on the other, sued one another for having given offence. The first court hearing took place on 11 May 1903. It did not differ much from the meeting at Seifert’s Saal: each of the parties cast aspersions on the other. The only aspect of the trial worth noting is its aftermath in front of the court building. Around a hundred *Volkssänger* gathered there and engaged in fierce debates about the events in the courtroom. When Adolf Hirsch, Albert’s son, caught sight of the lawyer representing his father’s adversaries, he hurled insults at him. In the heat of the moment, Adolf Hirsch even intended to attack him physically. Only the intervention of passers-by prevented a violent brawl (IWE 130, 12 May 1903, 11–12). The trial continued two weeks later. At this point, the judge succeeded in mediating a settlement between the defendants (IWE 144, 26 May 1903, 9).

The official end of the conflict in court, however, did not mean that the relationship between Hirsch and his detractors became harmonious. For many of his peers, Hirsch had become a *persona non grata* to be avoided. Hirsch’s career was adversely affected by this antipathy towards him. Not only did he fail to become director of the *Folies-Caprice*, he also faced severe difficulties in finding a venue for his own troupe to perform in the city. Consequently, he set out to tour neighbouring countries, but never ceased in his attempts to gain a foothold in Vienna (IWE 181, 1 July 1904; 15 and IWE 200, 20 July 1904, 9). He even promoted himself as an entertainer at private parties (IWE 44, 13 February 1904, 18). Over time, it becomes ever more difficult to keep track of his whereabouts. Apparently, did not continue performing in Vienna. The retribution of his former colleagues, who felt betrayed by Hirsch’s actions, had finally been achieved.

Albert Hirsch, a Jewish *Volkssänger*, questioned his colleagues’ nationalism and consequently lost his opportunities to perform. Admittedly, he did not condemn his colleagues’ anti-Hungarian sentiments in order to bring his own cosmopolitan ideals to bear. Rather, he did it for personal gain, thereby deceiving his peers. This improper behaviour was the major reason why many *Volkssänger* scorned him. In all likelihood, Hirsch’s Jewishness played no role in their disapproval of him: he was never the target of explicit anti-Semitism. Considering the historical context of Vienna around 1900, this is noteworthy (Pulzer 1988) After all, the city was one of the hotbeds of Judeophobia in Central Europe. Nevertheless, Hirsch enjoyed great respect among his colleagues. Over the years, he gained a reputation as an effective mediator between the *Volkssänger* and the government (IWE 78, 17 March 1896, 4). He was engaged in activities to help his destitute colleagues and served as an arbitrator in conflicts among them. On a personal level, he maintained ties with them and invited them to his home (IWE 130, 12 May 1903, 11–12). He truly was, it appears, one of them.

Not only Hirsch but also numerous other Jewish *Volkssänger* were spared Judeophobic vilification. The designation of the *Lemberger Singspiel-Gesellschaft* as “Polish Jews” was an unusual exception to the generally amicable relations among them. Another rare instance of biased thinking against Jewish performing musicians appeared in a newspaper article in September 1904. In the text, the author welcomes the appearance of a new Viennese song (*Wienerlied*). It is, he stresses, authentic, imbued with “spirit,” and full of folksy humour. He also claims that listening to this indigenous Viennese (“*urwienerisch”*) song provides relief after all the horrible songs that could be heard on stages in the recent past, after the *Mauscheln*, namely the Jewish mode of speaking, and Jewish stories, had taken over the stage (IWE 353, 24 December 1901, 9). In this note, Jews are depicted as unwarrantedly tampering with, and thereby defiling, Viennese traditions. They are seen as strangers to the city’s culture, unable to comprehend the sentiments of its inhabitants and thus to produce authentic Viennese songs.

The Viennese *Volkssänger* represented a milieu without clearly distinctive Jewish and non-Jewish realms. Indeed there were Jewish groups, but they also engaged non-Jews, just as non-Jewish ensembles included Jewish members (Hödl 2013, 389–391). Rather than difference, intermingling characterized the relations between Jewish and non-Jewish performing musicians. Consequently, a musician’s identification as Jewish or non-Jewish was difficult to uphold. In this context, Hirsch’s performative strategy was an adequate way to articulate his sense of belonging. He could thus be anything, depending on the situation in which he found himself. He was Viennese, Austrian, a *Volkssänger*, xenophobic, xenophilic and much more. Last but not least, he also was Jewish.

### Concluding remarks

One purpose of this essay was to introduce the novel concept of *similarity* into Jewish studies. Due to its abandonment of dichotomous classifications, in the particular case of Jews and non-Jews, and concurrent retention of differences between them, its application causes a shift in perspective on various aspects of Jewish history, draws attention to hitherto unexplored subjects and sheds new light on Jewish and non-Jewish interactions.

The second purpose was to give a short account of a conflict among performing musicians in Vienna in the early twentieth century. Its central figure, Albert Hirsch, was among the most popular Viennese *Volkssänger*. For a long time, his Jewishness was of no relevance to his colleagues. This changed when they felt deceived by him. Consequently, their prevalent anti-Hungarian attitude gave way to anti-Jewish sentiments. Some of the *Volkssänger* began to openly doubt their Jewish colleagues’ qualifications to compose and sing *Wienerlieder*, and thus to be included among their profession (See IWE 266, 25 September 1904, 18). Hirsch reacted to this development by questioning Jewish and non-Jewish demarcation lines. He did so by pointing out commonalities between himself and the anti-Semite Karl Lueger. In the light of given narratives, Hirsch’s line of argument must be considered odd. The application of similarity as an analytical tool, however, renders it comprehensible.

## Notes

1. This frequently happened when migrants planning to emigrate to the United States found they had insufficient funds for a steamer ticket or were barred from embarking for health reasons. See Howe (1976).

2. On Carl Lorens and Adolf Hirsch see Koller (1931, 115–117, 120 and 130–131).

3. In 1910, only 14.3% of Catholic men were independently employed, whereas more than 36% of the Jews were in this category. See Oxaal (1990, 58).

4. This dichotomy characterizes not only studies on anti-Semitism or Zionism, but Jewish mainstream historiography as well, as the use of ideas such as Jewish assimilation or acculturation indicate. See Blanchard (2002, 38).

5. See, for example, Bronner (2014).

6. The term *similarity* in italics refers to the specific conceptual understanding of this technical term.

7. The basic ideas of *similarity* were first elaborated by the French cultural sociologist Gabriel Tarde in 1890 (Tarde 2003).

8. Assmann quotes (Fromm 2001, 36).

9. This is, for example, what Mary Louise Pratt means by the term *contact zone*. See Pratt (1991, 33–40) and Pratt (1992, 1–11).

10. The emphasis on the transient indicates a borrowing of the model of similarity from concepts of the performative which, in their entirety, constitute the *performative turn*. On the understanding of performance in the context of cultural studies and the *performative turn*, see Fischer-Lichte (2003, 33–54); Fischer-Lichte (2004); and Fischer-Lichte (2001).

11. On the persistence of essentialized ideas of Jewish identity, see Glenn (2002, 139–152).

12. Kornberg Greenberg (1999, 16). For this reason, the term “identity” should even be abandoned and substituted by “identification”.

13. Newspapers described the conflicts among the *Volkssänger* as a war (“*Volkssängerkrieg*”) because of their intensity.

14. Hungary pursued a very nationalistic policy in that it banned all performances in German, not only those of Viennese *Volkssänger*. See IWE 160, 12 June 1902, 8.

15. At this time, some 60 *Volkssänger* ensembles were performing in Vienna, and each of them consisted of several members. See IWE 295, 26 Jan. 1902, 33.

16. About Karl Spacek (1850–1904) see IWE 161, 11 June 1904, 5–6.

17. Rötzer was one of the most important representatives of his profession. He was immensely productive. Around the turn of the twentieth century, he had already composed around one thousand songs, burlesques and short pieces. IWE (38, 8 February 1903, 4). See also Koller (1931, 145). Recher was a conductor and pianist (IWE 130, 12 May 1903, 12).

18. Hirsch was born in Vienna. Together with his wife and children, he formed his own ensemble (*Gesellschaft Hirsch*), which played in Yiddish. See Österreichisches Musiklexikon (Koller 1931, 131).

19. So-called *Damenkapellen* came into fashion around the mid-century and were an abiding component of the musical scene. Some of them even toured abroad, such as the Viennese Lady Orchestra, which performed in London in 1885 and initiated the establishment of such bands in England as well. See Scott (2008, 21–22).

20. On Amon Berg, see IWE 62, 3 March 1899, 5.

21. Sometimes, however, *tamburizza* and *Volkssänger* performances took place at the same location, the latter preceded by the concert. See IWE 179, 2 July 1903, 16.

22. The *Lemberger Singspiel-Gesellschaft* was founded in 1901. Most of its members were former actors of the Yiddish theatre in Lemberg (today Lviv in Ukraine). See Karner (2005, 118). Little is known about the *Folies-Comiques*. Some references can be found in Schäffer (2008, 18).

23. The *Budapester Orpheumgesellschaft* was the most important Jewish *Volkssänger* ensemble. See Wacks (2002).

24. IWE 74, 16 March 1903, 13: “A. Hirsch, Wiener und eventueller alleiniger Director des neues Wiener Familien-Variété im ‘Hotel Central.’”

25. In this context, the term “performance” stresses acting as opposed to “being,” the dynamic over against the static.

26. “In der Kirche ist neben dem Juden Hirsch der Bürgermeister gestanden. Sie können nicht verlangen, meine Herren, dass ich ‘Hoch Lueger’ rufe, ich bin Jude, aber es war doch schön von ihm, dass er da war. Wer hat bei diesem Fest gefehlt? Die Budapester.”(see *Das Variété* 21, 25 March 1901, n.p.).

## References

[CIT0001] Assmann Aleida. 2015 “Ähnlichkeit als Performanz. Ein Neuer Zugang zu Identitätskonstruktionen und Empathie-Regimen.” In *Ähnlichkeit. Ein Kulturtheoretisches Paradigma*, edited by Bhatti Anil and Kimmich Dorothee, 167–185. Konstanz: Konstanz University Press.

[CIT0002] Augé Marc. 2014 *Nicht-Orte*. Munich4: C. H. Beck.

[CIT0003] Berkley George E. 1988 *Vienna and Its Jews: The Tragedy of Success, 1880s-1980s*. Boston, MA: Madison Books.

[CIT0004] Bhatti Anil. 2016 “>Ähnlichkeit</>Plurikulturalität<. Vorläufige Überlegungen.” In *Habsburg neu Denken. Vielfalt und Ambivalenz in Zentraleuropa. 30 Kulturwissenschaftliche Stichworte*, edited by Feichtinger Johannes and Uhl Heidemarie, 171–180. Vienna: Böhlau Verlag.

[CIT0005] Brauner-Berger Elisabeth. 1993 “Volkssängertum im Wandel.” PhD thesis. Unpubl.

[CIT0006] Blanchard Tsvi. 2002 “How to Think About Being Jewish in the Twenty-first Century: A New Model of Jewish Identity Construction”. *Journal of Jewish Community Service* (Fall) 79 (1): 38.

[CIT0007] Bohlman Philip V. 2008 *Jewish Music and Modernity*. Oxford: Oxford University Press.

[CIT0008] Boyer John W. 1981 *Political Radicalism in Late Imperial Vienna*. Chicago: The University of Chicago Press.

[CIT0009] Bronner Simon J., ed. 2014 *Framing Jewish Culture: Boundaries and Representations*. Oxford: The Littman Library of Jewish Civilization.

[CIT0010] Burákova Zuzana. 2016 “The Elusive Character of American Jewish Identity”. Accessed November 4, 2016. http://www.pulib.sk/elpub2/FF/Ferencik2/pdf_doc/4.pdf.

[CIT0011] Das Variété 20, 18 March 1903, n.p.

[CIT0011a] Das Variété 21, 25 March 1903, n.p.

[CIT0013] Fischer-Lichte Erika. 2004 *Ästhetik des Performativen*. Frankfurt/M: edition suhrkamp.

[CIT0014] Fischer-Lichte Erika. 2001 *Ästhetische Erfahrung. Das Semiotische und das Performative*. Tübingen: Francke.

[CIT0015] Fischer-Lichte Erika. 2003 “Performance, Inszenierung, Ritual. Zur Klärung kulturwissenschaftlicher Schlüsselbegriffe.” In *Geschichtswissenschaft und “performative turn”. Ritual, Inszenierung und Performanz vom Mittelalter bis zur Neuzeit*, edited by Martschukat Jürgen and Steffen Patzold, 33–54. Cologne: Böhlau.

[CIT0016] Foucault Michel. 2014 *Die Heterotopien. Der utopische Körper. Zwei Radiovorträge*. Frankfurt/M.2: Suhrkamp taschenbuch wissenschaft.

[CIT0017] Fromm Waldemar. 2001 “Die Sympathie des Zeichens mit dem Bezeichneten. Ähnlichkeit in Literatur und Sprachästhetik um 1800.” In *Ästhetik der Ähnlichkeit. Zur Poetik und Kunstphilosophie der Moderne*, edited by Funk Gerald, Mattenklott Gert and Pauen Michael, 35–68. Frankfurt/M: Fischer 2001.

[CIT0018] Geehr Richard S. 2003 *The Aesthetics of Horror: The Life and Thought of Richard von Kralik*. Boston: Brill Academic.

[CIT0019] Gennep Arnold van. 1999 *Übergangsriten (Les rites de passage)*. Frankfurt/M: Campus.

[CIT0020] Glenn Susan A. 2002 “In the Blood? Consent, Descent, and the Ironies of Jewish Identity.” *Jewish Social Studies: History, Culture, and Society* 8 (2/3): 139–152. doi: 10.2979/JSS.2002.8.2-3.139

[CIT0021] Gluck Mary. 2016 *The Invisible Jewish Budapest. Metropolitan Culture at the Fin de Siècle*. Madison: The University of Wisconsin Press.

[CIT0022] Hauenstein Hans. 1976 *Chronik des Wienerliedes. Ein Streifzug von den Minnesängern über den Lieben Augustin, den Harfenisten und Volkssängern bis in die heutige Zeit*. Klosterneuburg: Jasomirgott-Verlag.

[CIT0023] Hödl Klaus, 1993 *Als Bettler in die Leopoldstadt. Galizische Juden auf dem Weg Nach Wien*. Vienna: Böhlau Verlag.

[CIT0024] Hödl Klaus. 1999 “Galician Jewish Migration to Vienna.” *Polin* 12: 147–163.

[CIT0025] Hödl Klaus. 2013 “The Elusiveness of Jewishness: Jews in Viennese Popular Culture Around 1900.” *Journal of Modern Jewish Studies* 12 (3): 379–397. doi: 10.1080/14725886.2013.828897

[CIT0026] Howe Irving. 1976 *The World of Our Fathers*. New York: Schocken.

[CIT0027] Illustrirtes Wiener Extrablatt (hereafter: IWE) 78 17 March 1896 4.

[CIT0028] IWE 23 23 January 1897 8.

[CIT0029] IWE 62 3 March 1899 5.

[CIT0030] IWE 183 6 July 1900 15.

[CIT0031] IWE 283 15 October 1900 3.

[CIT0032] IWE 358 31 December 1900 2–3.

[CIT0033] IWE 350 21 December 1901 15.

[CIT0034] IWE 353 24 December 1901 9.

[CIT0035] IWE 356 26 December 1901 6.

[CIT0036] IWE 356 28 December 1901 4.

[CIT0037] IWE 295 26 January 1902 33.

[CIT0038] IWE 160 12 June 1902 8.

[CIT0039] IWE 38 8 February 1903 4.

[CIT0040] IWE 130 12 May 1903 12.

[CIT0041] IWE 179 2 July 1903 16.

[CIT0042] IWE 74 16 March 1903 3.

[CIT0043] IWE 74 16 March 1903 13.

[CIT0044] IWE 130 12 May 1903 11–12.

[CIT0045] IWE 144 26 May 1903 9.

[CIT0046] IEW 44 13 February 1904 18.

[CIT0047] IWE 161 11 June 1904 5–6.

[CIT0048] IWE 181 1 July 1904 15.

[CIT0049] IWE 200 20 July 1904 9.

[CIT0050] IWE 266 25 September 1904 18.

[CIT0051] IWE 53 23 February 1907 7.

[CIT0052] Karner Doris A. 2005 *Lachen unter Tränen. Jüdisches Theater in Ostgalizien und der Bukowina*. Vienna: Edition Steinbauer.

[CIT0053] Koller Josef 1931 *Das Wiener Volkssängertum in alter und neuer Zeit. Nacherzähltes und Selbsterlebtes*. Vienna: Gerlach & Wiedling.

[CIT0054] Kornberg Greenberg Yudit. 1999 “The Choosing, not the Chosen People.” In *Jewish Identity in the Postmodern Age*, edited by Selengut Charles, 13–24. St. Paul: Paragon House.

[CIT0055] Koschorke Albrecht. 2015 “Valenzen eines postkolonialen Konzepts.” In *Ähnlichkeit. Ein kulturtheoretisches Paradigma*, edited by Bhatti Anil and Kimmich Dorothee, 35–45. Konstanz: Konstanz University Press.

[CIT0056] Langenohl Andreas. 2015 “Ähnlichkeit als differenztheoretisches Konzept: Zur Reformulierung der Modernisierungstheorie.” In *Ähnlichkeit. Ein kulturtheoretisches Paradigma*, edited by Bhatti Anil, 105–127.

[CIT0057] Lappin Eleonore. 2009 “Jüdische Lebenserinnerungen. Rekonstruktionen von jüdischer Kindheit und Jugend im Wien der Zwischenkriegszeit.” In *Wien und die jüdische Erfahrung 1900–1938. Akkulturation, Antisemitismus, Zionismus*, edited by Stern Frank and Eichinger Barbara, 17–38. Vienna: Böhlau Verlag.

[CIT0058] Latour Bruno. 2014 *Eine neue Soziologie für eine neue Gesellschaft. Einführung in die Akteur-Netzwerk-Theorie*. Frankfurt/M: 3 suhrkamp taschenbuch wissenschaft.

[CIT0059] Leitner Carola, and Hamtil Kurt 2006 *Ottakring. Wiens 16. Bezirk in alten Fotografien*. Vienna: Verlag Carl Ueberreuter.

[CIT0060] Maderthaner Wolfgang, and Musner Lutz 1999 *Die Anarchie der Vorstadt. Das andere Wien um 1900*. Frankfurt/M: Campus Verlag.

[CIT0061] Osterhammel Jürgen. 2015 “Ähnlichkeit – Divergenz – Konvergenz. Für eine Historiographie relationaler Prozesse.” In *Ähnlichkeit. Ein kulturtheoretisches Paradigma*, edited by Bhatti Anil and Kimmich Dorothee, 75–91. Konstanz: Konstanz University Press.

[CIT0063] Oxaal Ivar. 1990 “Die Juden im Wien des jungen Hitler: Historische und soziologische Aspekte.” In *Eine zerstörte Kultur. Jüdisches Leben und Antisemitismus in Wien seit dem 19. Jahrhundert*, edited by Botz Gerhard, Oxaal Ivar and Pollak Michael, 29–65. Buchloe: Verlag Obermayer.

[CIT0064] Pauley Bruce. 1992 *From Prejudice to Persecution: A History of Austrian Anti-Semitism*. Chapel Hill: The University of North Carolina Press.

[CIT0065] Penslar Derek. 2009 “The New Face of German-Jewish Historiography: Comparative, Trans-National, International.” *Leo Baeck Institute Yearbook* 54 (2009): 10–14.

[CIT0066] Perry Micha J., and Voß Rebekka 2016 “Approaching Shared Heroes: Cultural Transfer and Transnational Jewish History.” *Jewish History* 30 (2016): 1–13. doi: 10.1007/s10835-016-9253-x

[CIT0067] Pratt Mary Louise. 1991 “Arts of the Contact Zone.” *Profession*, 33–40.

[CIT0068] Pratt Mary Louise. 1992 “Introduction: Criticism in the Contact Zone.” In *Imperial Eyes: Travel Writing and Transculturation*, edited by Pratt Mary Louise, 1–11. New York: Routledge.

[CIT0069] Pulzer Peter. 1988 *The Rise of Political Anti-Semitism in Germany & Austria*. Cambridge, MA: Harvard University Press.

[CIT0070] Rosman Moshe. 2007 *How Jewish is Jewish History?* Oxford: The Littman Library of Jewish Civilization.

[CIT0071] Scott Derek B. 2008 *Sounds of the Metropolis: The Nineteenth-century Popular Music Revolution in London, New York, Paris, and Vienna*. Oxford: Oxford University Press.

[CIT0072] Schäffer Verena. 2008 “Theater und Varieté im Nestroyhof: 1899 bis 1938. Vier Jahrzehnte Theatraler Produktion in der Wiener Leopoldstadt.” PhD thesis. Unpubl.

[CIT0073] Schmale Wolfgang and Steer Martina, eds. 2006 *Kulturtransfer in der jüdischen Geschichte*. Frankfurt/M: Campus Verlag.

[CIT0074] Tarde Gabriel. 2003 *Die Gesetze der Nachahmung*. Frankfurt/M: Suhrkamp.

[CIT0075] Torberg Friedrich. 2011 “Warum ich darauf stolz bin.”*Jüdischer Almanach* Sport: 59–68.

[CIT0076] Turner Victor. 2000 *Das Ritual. Struktur und Anti-Struktur*. Frankfurt/M: Campus Verlag.

[CIT0077] Wacks Georg. 2002 *Die Budapester Orpheumgsellschaft. Ein Varieté in Wien 1889–1919*. Vienna: Verlag Holzhausen.

